# Baseline Cardiovascular Risk Factors and Stroke Mortality by Municipality Population Size in a 19-year Follow-up Study-NIPPON DATA80

**DOI:** 10.2188/jea.JE2008034

**Published:** 2008-08-07

**Authors:** Nobuo Nishi

**Affiliations:** 1 Department of Epidemiology, Hiroshima Laboratory, Radiation Effects Research Foundation

**Keywords:** cardiovascular risk factors, stroke mortality, urban population, rural population, multilevel analysis

## Abstract

The urban-rural difference in cardiovascular risk factors and stroke mortality throughout Japan was examined in a cohort by using hierarchical data structure. The subjects were 9,309 men and women aged ≥ 30 years who were residents of 294 areas in 211 municipalities of Japan in 1980; they were followed up until 1999. The population sizes of the municipalities in which the aforementioned areas were located were used to distinguish between urban and rural areas. We applied multilevel modeling to take into account the hierarchical data structure of individuals (subjects) (level 1) nested within areas (level 2). Statistically significant differences were observed in the case of medium (30,000-300,000) and small (<30,000) municipality populations compared with large (≥300,000) municipality populations with regard to the following parameters: body mass index in men, serum total cholesterol in both men and women, and daily alcohol drinking in women. The values or frequencies of these cardiovascular risk factors were significantly higher in large populations. Meanwhile, age-adjusted odds ratios for stroke mortality in the areas in the medium and small municipalities compared with those in the areas in the large municipalities were 1.31 (95% confidence interval (CI) 0.81-2.13) and 1.40 (95% CI 0.87-2.24) in men, and 1.32 (95% CI 0.79-2.20) and 1.62 (95% CI 0.99-2.65) in women, respectively. The results of multivariate analyses adjusted for age, body mass index, total cholesterol, diabetes, hypertension, current smoking, and daily alcohol consumption did not change materially. In conclusion, stroke mortality tended to be higher in rural areas than in urban areas in Japan, especially among women.

## INTRODUCTION

From the 1950s to the 1970s, stroke was the leading cause of death in Japan.^1^ Geographical variation in stroke mortality was first recognized in the 1950s, and standardized mortality ratios for cerebral hemorrhage and cerebral infarction were found to be high in the northeastern part of the country, especially the Tohoku region.^2^^-^^4^ Stroke mortality started to decline in the 1970s as a result of community efforts such as the detection and control of hypertension and the reduction of dietary sodium intake.^5^^-^^8^ Cohort studies on stroke have mainly focused on rural areas, with few studies comparing stroke mortality between urban and rural areas in Japan. From 1964 to 1995, Kitamura et al. conducted cohort studies in a rural town in the Tohoku region and in an urban community in western Japan and reported that stroke mortality in the rural town was consistently higher than that in the urban community, especially among younger men aged 40-59 years.^9^ However, their comparison was based on data from 2 areas in vastly different regions and did not reflect the overall difference in stroke mortality between urban and rural areas in Japan.

The National Integrated Project for Prospective Observation of Non-communicable Diseases And its Trends in the Aged, 1980 (NIPPON DATA80) is suitable for exploring urban-rural differences in stroke mortality throughout Japan because of its hierarchical data structure. This paper shows the urban-rural difference in stroke mortality over 19 years of follow-up conducted as a part of NIPPON DATA80 as well as the baseline cardiovascular risk factors.

## NIPPON DATA80 AND MUNICIPALITY POPULATION SIZE

### Study population

NIPPON DATA80 is an observational cohort study on a representative sample of the general Japanese population aged 30 years or older, with a 19-year follow-up conducted over the last 2 decades of the 20th century; the details of which have been reported elsewhere.^10^^-^^12^ In brief, 10,546 community residents (4,640 men and 5,906 women) aged 30 years or older from 300 randomly selected areas participated in the National Survey on Circulatory Disorders 1980.

### Survey area and administrative boundary of Japan

Each survey area was located in a public health center district, and the chief of the public health center was responsible for the conduct of the survey. Public health centers have been founded by all 47 prefectures and designated cities (municipalities with large populations) since 1937 and by special wards in the Tokyo Metropolitan Area since 1946, and these have played a pivotal role in the administration of public health in Japan. The establishment of a public health center is closely related to the municipality population size. A public health center founded by a prefecture might include several small municipalities such as villages, towns, and (small) cities, whereas a designated city or special ward might include 1 or more public health centers. Thus, a public health center district is designated either by combining small municipalities or by dividing large municipalities, to ensure that each public health center covers approximately 100,000 people. A total of 855 public health centers and about 3,250 municipalities existed in Japan in 1980. The 300 study areas in our study were located in 300 public health center districts in 211 municipalities.

The data were characterized using a three-level structure: individuals at level 1 were nested within areas at level 2, which in turn were nested within regions at level 3. Regions (level 3) were defined by dividing Japan’s 47 prefectures into 6 groups: Hokkaido-Tohoku (7 prefectures), Kanto-Koshin (9), Hokuriku-Tokai (8), Kinki (6), Chugoku-Shikoku (9), and Kyushu (8). Areas (level 2) were classified as urban or rural based on the population size of the municipality in which they were located. As mentioned above, each survey area was located in a public health center district, but the population size of the municipality proved to be more reliable for defining a given area as urban or rural. This was because municipalities in Japan are designated by the government as *mura* (village), *machi* (town), or *shi* (city) according to their level of development (special wards in the Tokyo Metropolitan Area are called *ku*), and a key determinant of this classification is population size. In our study, 294 areas in the 6 regions were categorized into 3 groups according to the population size of the municipalities: small municipality with less than 30,000 people, medium municipality with 30,000-300,000 people, and large municipality with 300,000 or more people. These categories were used because a population of 30,000 is required for a city designation, while a population of 300,000 is required for a core city designation, in accordance with Japan’s Local Autonomy Law.

### Endpoint determination

The procedure used for endpoint determination has been reported elsewhere.^10^^-^^12^ In brief, the underlying causes of death reported in Japan’s National Vital Statistics were coded according to the 9th International Classification of Disease (ICD-9) until the end of 1994 and the ICD-10 from the beginning of 1995. Codes 430-434 and 436-438 in ICD-9 and I60-I69 in ICD-10 were defined as death from total stroke, which included death from cerebral infarction (codes 433, 434, 437.7a, and 7b in ICD-9, and I63 and I69.3 in ICD-10) and from cerebral hemorrhage (codes 431 and 432 in ICD-9, and I61 and I69.1 in ICD-10).

The present study is based on the outcome information obtained until November 1999, making the observation period 19 years long. Of the 10,546 participants, 1,237 were excluded because of past history of coronary heart disease or stroke at baseline survey (n = 280), missing information at baseline survey (n = 84), or designation of “lost to follow-up” due to failed efforts to locate certain subjects (n = 873). This left 9,309 participants (4,080 men and 5,229 women) from 294 areas in 211 municipalities.

The numbers and percentages of areas and subjects by municipality population size in the 6 regions are shown in Table 1. The numbers of municipalities with large, medium, and small population sizes were 40, 104, and 67, respectively, while the numbers of areas in these municipalities were 114, 113, and 67, respectively. This information indicates that municipalities with large population size had 1 or more public health center. The percentages of municipalities with large, medium, and small population sizes differed significantly by region (*P* < 0.001). The regions were listed from the northeastern (Hokkaido-Tohoku) to the southwestern (Kyushu) part of the Japanese archipelago, and the percentage of municipalities with large population size was lower in the Hokkaido-Tohoku region and higher in the Kanto-Koshin and Kinki regions.

**Table 1.** Numbers and percentages of areas (level 2) and subjects (level 1) in 6 regions (level 3) by municipality population size (Japanese men and women aged 30 years and older in 1980, NIPPON DATA80)

Regions (level 3)	Areas (level 2) and subjects (level 1)	Municipality population size (n = 211)						Total	

		Large (n = 40)		Medium (n = 104)		Small (n = 67)			
		No.	%	No.	%	No.	%	No.	%
Hokkaido-Tohoku	Areas	4	11.4	18	51.4	13	37.1	35	100.0
	*Subjects*	*79*	*5.8*	*596*	*43.9*	*682*	*50.3*	*1,357*	*100.0*
Kanto-Koshin	Areas	44	47.8	34	37.0	14	15.2	92	100.0
	*Subjects*	*784*	*34.1*	*826*	*35.9*	*691*	*30.0*	*2,301*	*100.0*
Hokuriku-Tokai	Areas	15	30.0	23	46.0	12	24.0	50	100.0
	*Subjects*	*480*	*22.9*	*930*	*44.3*	*689*	*32.8*	*2,099*	*100.0*
Kinki	Areas	27	51.9	20	38.5	5	9.6	52	100.0
	*Subjects*	*549*	*41.5*	*564*	*42.6*	*210*	*15.9*	*1,323*	*100.0*
Chugoku-Shikoku	Areas	10	34.5	7	24.1	12	41.4	29	100.0
	*Subjects*	*319*	*31.2*	*240*	*23.5*	*464*	*45.4*	*1,023*	*100.0*
Kyushu	Areas	14	38.9	11	30.6	11	30.6	36	100.0
	*Subjects*	*330*	*27.4*	*406*	*33.7*	*470*	*39.0*	*1,206*	*100.0*
Total	Areas	114	38.8	113	38.4	67	22.8	294	100.0
	*Subjects*	*2,541*	*27.3*	*3,562*	*38.3*	*3,206*	*34.4*	*9,309*	*100.0*

Municipality population size: large (≥300,000), medium (30,000-300,000), and small (<30,000)

### Statistical analysis

To illustrate the model of a three-level structure with individuals at level 1 nested within areas at level 2 that were nested within regions at level 3, we used multilevel regression procedures.^13^ For a binary variable, we used multilevel logistic regression on a logit-link function. Models were fitted using MLwiN software, version 2.0.^14^ The results were expressed as regression coefficients and standard error, and odds ratios and 95% confidence intervals (CIs). The significance of the regression coefficients was tested using Wald statistic. Intraclass correlation coefficient was estimated from the two-level null random intercept model as r_0_^2^/(r_0_^2^ + 3.29), where r_0_^2^ is the unexplained random variance at level 2.^15^

## CARDIOVASCULAR RISK FACTORS BY MUNICIPALITY POPULATION SIZE

### Baseline characteristics

Table 2 shows the baseline characteristics of the subjects by municipality population size. Body mass index was calculated by dividing the weight (kg) by the square of the height (m). Diabetes was defined as a serum glucose level of 11.1 mmol/l or higher, history of diabetes, or both. Hypertension was defined as systolic blood pressure of 140 mmHg or higher, diastolic blood pressure of 90 mmHg or higher, use of antihypertensive agents, or any combination of these.

**Table 2.** Baseline characteristics of subjects by municipality population size (Japanese men and women aged 30 years and older in 1980, NIPPON DATA80)

	Municipality population size		

	Large	Medium	Small
Men			
Number of subjects	1,082	1,570	1,428
Age (y)	49.0 (12.6)	49.5 (12.9)	52.1 (13.6)
Body mass index (kg/m^2^)	22.8 (3.0)	22.5 (2.9)	22.3 (2.7)
Serum total cholesterol (mmol/l)	191.1 (32.5)	186.9 (32.9)	181.5 (32.2)
Diabetes (%)	7.1	6.1	7.7
Hypertension (%)	47.0	49.2	52.9
Current smoking (%)	60.4	65.7	62.7
Daily alcohol drinking (%)	48.0	47.1	49.5
Women			
Number of subjects	1,459	1,992	1,778
Age (y)	48.8 (12.8)	50.2 (13.1)	53 (13.6)
Body mass index (kg/m^2^)	22.7 (3.3)	22.8 (3.5)	23 (3.3)
Serum total cholesterol (mmol/l)	193.2 (34.5)	191.1 (34.2)	188.5 (33.5)
Diabetes (%)	3.8	4.5	4.0
Hypertension (%)	37.6	40.8	44.7
Current smoking (%)	11.7	9.4	5.7
Daily alcohol drinking (%)	4.2	2.7	1.9

Municipality population size: large (≥300,000), medium (30,000-300,000), and small (<30,000)

Diabetes was defined as a serum glucose level ≥ 11.1 mmol/l, history of diabetes, or both

Hypertension was defined as systolic blood pressure ≥ 140 mmHg, diastolic blood pressure ≥ 90 mmHg, or antihypertensive drug use

The subjects in the small populations were significantly older, both men and women, as determined using analysis of variance (ANOVA) (*P* < 0.001). Differences in the levels of cardiovascular risk factors according to the municipality population size were not tested using two-way ANOVA because they were tested in a subsequent two-level multilevel analysis (Table 3).

**Table 3.** Regression coefficients (SE) of cardiovascular risk factors by municipality population size assessed using two-level multilevel logistic regression analysis at the baseline of a 19-year follow-up of Japanese men and women aged 30 years and older in 1980 (NIPPON DATA80)

	Body mass index (kg/m^2^)	Serum total cholesterol (mg/dl)	Diabetes (yes/no)	Hypertension (yes/no)	Current smoking (yes/no)	Daily alcohol drinking (yes/no)
Men						
Fixed parameter						
Individual level						
Age	-0.03 (0.003)**	-0.09 (0.04) *	0.04 (0.005) **	0.06 (0.003) **	-0.02 (0.003) **	-0.006 (0.002) **
Area level						
Municipality population size						
Medium	-0.29 (0.13) *	-3.82 (1.70) *	-0.19 (0.16)	0.05 (0.10)	0.23 (0.09) *	-0.03 (0.09)
Small	-0.33 (0.14) *	-9.62 (1.82) **	-0.04 (0.16)	0.08 (0.11)	0.17 (0.10)	0.08 (0.10)
Random parameter						
Between areas	0.23 (0.07) **	62.7 (11.5) **	0.06 (0.09)	0.14 (0.04) **	0.08 (0.03) **	0.09 (0.03) **
Women						
Fixed parameter						
Individual level						
Age	0.01 (0.004) **	0.79 (0.03) **	0.05 (0.005) **	0.08 (0.003) **	0.01 (0.004) *	0.02 (0.006) **
Area level						
Municipality population size						
Medium	0.13 (0.15)	-3.29 (1.51) *	0.12 (0.19)	0.03 (0.11)	-0.25 (0.14)	-0.49 (0.21) *
Small	0.26 (0.16)	-8.64 (1.62) **	-0.18 (0.20)	-0.03 (0.11)	-0.81 (0.16) **	-0.90 (0.24) **
Random parameter						
Between areas	0.45 (0.09) **	51.3 (9.18) **	0.15 (0.12)	0.22 (0.04) **	0.27 (0.08) **	0.31 (0.18)

Municipality population size: large (≥300,000), medium (30,000-300,000), and small (<30,000)

*P < 0.05 and **P < 0.01 (Wald test)

### Results of multilevel analysis

Table 3 shows the regression coefficients of the cardiovascular risk factors at the baseline, as determined using two-level multilevel analysis. Age at individual level and municipality population size at area level were entered into the models as fixed parameters. Statistically significant variance between areas was observed in the following parameters: body mass index (kg/m^2^), serum total cholesterol (mg/dl), hypertension (yes/no), current smoker (yes/no) for both men and women, and daily alcohol drinker (yes/no) for men. Age (years) was significantly associated with all the cardiovascular risk factors examined. In the medium and small municipality populations versus the large municipality populations, statistically significant differences were observed in the following: body mass index in men, serum total cholesterol in men and women, and daily alcohol drinking in women. Negative regression coefficients indicated that the values or frequencies of these cardiovascular risk factors were significantly higher among the subjects in the municipalities with large populations. Regarding current smokers, a significant positive regression coefficient was observed among men in municipalities with medium population sizes, and a significant negative regression coefficient was observed among women in municipalities with small population sizes, indicating that the percentage of male current smokers was high in the former and that of female current smokers, low in the latter. Thus, a clear inverse relationship was not observed between the baseline cardiovascular risk factors of NIPPON DATA80 and municipality population size.

Fukuda et al. demonstrated that after adjustments for individual socioeconomic status, higher per capita income was significantly associated with current smoking and excessive alcohol drinking in women, as determined using multilevel analysis of the data from the 2001 Comprehensive Survey of the Living Conditions of People on Health and Welfare.^16^ It seems that the same tendency persisted for at least 20 years after the baseline survey of the NIPPON DATA80.

## STROKE MORTALITY BY MUNICIPALITY POPULATION SIZE

### Crude and age-adjusted mortality rates

Table 4 shows the numbers of persons, person-years, numbers of deaths, and mortality rate from total stroke categorized by municipality population size. In both men and women, the crude mortality rates were higher in municipalities with smaller population sizes, but this tendency was not observed in the case of age-adjusted mortality rate in men. Age-adjusted mortality rate from total stroke was highest among men in large municipalities; however, the age-specific mortality rate in the oldest age group, i.e., ≥85 years, markedly increased the overall age-adjusted mortality rate (only 1 person belonged to this age group, and he died of a stroke after a relatively short time). When the age groups 80-84 years and ≥85 years were combined, the age-adjusted mortality rates from total stroke in municipalities with large, medium, and small population sizes were 1.3, 1.5, and 1.6 in men and 0.8, 0.9, and 1.1 in women, respectively.

**Table 4.** Numbers of persons, person-years, and deaths; and crude and age-adjusted mortality rates from total stroke categorized by municipality population size in a 19-year follow-up of Japanese men and women aged 30 years and older in 1980 (NIPPON DATA80)

Municipality population size	No. of persons	No. of person-years	No. of deaths from total stroke	Mortality rate from total stroke (per 1,000)	

				Crude	Age-adjusted
Men					
Large	1,082	18,719	30	1.6	2.4
Medium	1,570	26,869	59	2.2	1.4
Small	1,428	23,502	73	3.1	1.7
Women					
Large	1,459	26,086	25	1.0	0.9
Medium	1,992	35,196	52	1.5	1.1
Small	1,778	30,464	73	2.4	1.3

Municipality population size: large (≥300,000), medium (30,000-300,000), and small (<30,000)

Age-adjusted mortality rate was standardized in accordance with the world population

### Results of multilevel analysis

In the three-level model where 6 regions of Japan were entered at level 3, no variance was observed at level 3, and the parameters at level 2 (areas) and level 1 (individuals) differed only slightly from those in the two-level model (individuals at level 1 nested within areas at level 2). The results of the two-level multilevel analyses are therefore shown in Table 5.

**Table 5.** Regression coefficients and odds ratios of deaths from total stroke categorized by municipality population size and determined using two-level multilevel logistic regression analysis in 19-year follow-up of Japanese men and women aged 30 years and older in 1980 (NIPPON DATA80)

	Age-adjusted (Model 2)		Multivariate-adjusted (Model 3)	
	
	Regression coefficients (SE)	Odds ratios (95% CI)	Regression coefficients (SE)	Odds ratios (95% CI)
Men				
Fixed parameters				
Individual level				
Age	0.10 (0.01)	1.11 (1.09-1.12)	0.10 (0.01)	1.11 (1.09-1.12)
Area level				
Municipality population size				
Medium	0.27 (0.25)	1.31 (0.81-2.13)	0.26 (0.25)	1.29 (0.80-2.10)
Small	0.33 (0.24)	1.40 (0.87-2.24)	0.30 (0.24)	1.36 (0.84-2.18)
Random parameters				
Between areas	0.15 (0.15)		0.12 (0.15)	
Women				
Fixed parameters				
Individual level				
Age	0.11 (0.01)	1.11 (1.10-1.13)	0.10 (0.01)	1.11 (1.09-1.12)
Area level				
Municipality population size				
Medium	0.28 (0.26)	1.32 (0.79-2.20)	0.29 (0.26)	1.34 (0.80-2.23)
Small	0.48 (0.25)	1.62 (0.99-2.65)	0.52 (0.25)	1.68 (1.02-2.77)
Random parameters				
Between areas	0.09 (0.15)		0.10 (0.15)	

Municipality population size: large (≥300,000), medium (30,000-300,000), and small (<30,000)

Model 1, null model (data not shown); Model 2, age-adjusted; and Model 3, adjusted for age, body mass index, serum total cholesterol, diabetes, hypertension, current smoking, and daily alcohol drinking.

The following variables were added successively to the null model (Model 1), municipality population size (medium and small population sizes compared with large population size), age (Model 2), and body mass index, serum total cholesterol, diabetes (serum glucose ≥ 11.1 mmol/l, history of diabetes, or both), hypertension (systolic blood pressure ≥ 140 mmHg, diastolic blood pressure ≥ 90 mmHg, or antihypertensive drug use), current smoking, and daily alcohol consumption (Model 3). In Model 1 (null model), statistically significant variance between areas was not observed in men (*P* = 0.12) but was observed in women (*P* = 0.04). The intraclass correlation coefficients were 7.3% and 10.6% in men and women respectively. In the age-adjusted model (Model 2) and the multivariate-adjusted model (Model 3), women had higher regression coefficients for municipality population sizes. Odds ratios and 95% CIs were calculated in the age-adjusted and the multivariate-adjusted models and significantly elevated odds ratio for small population size compared with large population size was observed for women in the multivariate-adjusted model. In multivariate analyses, we used a dichotomous variable for hypertension. We then analyzed the data using a continuous variable for systolic and diastolic blood pressure, but similar results were obtained.

As our study areas were randomly selected from across Japan, the results are considered to reflect the general urban-rural difference in stroke mortality in Japan. As no variance was found between regions (level 3) in the three-level model, the geographical variation in stroke mortality reported a few decades ago in several ecological studies^2^^-^^4^ might have been partly a reflection of the unbalanced distribution of urban and rural areas by region. This is a plausible explanation because the distributions of municipalities by population size were significantly different (Table 1), and the northeastern part of Japan in particular was characterized as a region with a higher proportion of municipalities with small population sizes, that is, rural areas.

The urban-rural difference in total stroke was more pronounced in women than in men. To examine these results in further detail, we also analyzed the data for cerebral infarction, which is a dominant stroke type in our cohort. Although statistically significant variance in the two-level null model was observed only among women, the odds ratios of deaths from cerebral infarction in the municipalities with medium and small population sizes compared with those in the municipalities with large population sizes were higher than unity only among women.^17^ This contrast between men and women in the urban-rural difference in cerebral infarction-related deaths seems to have contributed to the gender difference in the urban-rural gradient.

Gender difference was also observed in the difference in the odds ratios for total stroke between the age-adjusted and the multivariate-adjusted models. Multivariate analyses revealed that the urban-rural difference in stroke mortality persisted after adjustment for risk factors, and statistically significant risk factors other than age were hypertension and current smoking in men and hypertension in women. The odds ratios in the multivariate-adjusted model by municipality population size were slightly lower in men and slightly higher in women. This result may be attributable to the fact that smoking was differently associated with municipality population sizes in the case of men and women. Among the baseline characteristics, body mass index in men and serum total cholesterol in both men and women were statistically positively associated with municipality population size, but they were neither positively or negatively associated with the risks of total stroke or cerebral infarction.

Differences in medical resources might also have affected the urban-rural difference in stroke mortality in the study. For many years rural areas faced a lack of medical resources, and to remedy this shortage, many medical schools were founded throughout Japan in the 1980s. However, Kobayashi and Takaki revealed that a doubling of the number of physicians due to the increased number of medical schools failed to improve the disproportionate urban-rural distribution of physicians.^18^ It is not surprising that areas with fewer medical resources are disadvantaged with respect to the early detection and treatment of stroke.

### Findings in other countries

Stroke incidence and mortality have been compared between urban and rural areas in several countries.^19^^-^^23^ Some studies have reported higher rates in rural areas.^19^^,^^21^^,^^22^ Others have reported similar rates in the 2 areas^23^ or even lower rates in rural areas.^20^ Correia et al. reported higher stroke incidence among a rural population compared with that in an urban population in northern Portugal.^22^ In Shanghai, China, the stroke incidence among rural men and the mortality rate among rural men and women aged 65-74 years were higher than those of their urban counterparts in the period from 1984 to 1991.^19^ As our data only involved stroke mortality, it is necessary to further investigate the difference in stroke incidence and case fatality between urban and rural areas.

### Limitations

There were some limitations to our study. First, individual socioeconomic factors were not taken into account because data for income and level of education were not available and data for occupation were limited to working subjects. Second, the use of Japan’s National Vital Statistics for stroke deaths where stroke subtypes may generally be misclassified on death certificates is considered a possible shortcoming. However, most stroke cases in Japan are referred to hospitals. Moreover, computerized tomography scanning was performed in over 85% of stroke patients in the 1980s, even in the rural areas, throughout Japan.^24^ Finally, we used a multilevel logistic regression model that did not take observation period into consideration. When we applied a frailty model of Cox regression,^25^ hazard ratios for the medium and small population sizes compared with the large population size were slightly higher than the respective odds ratios from the present study, but the results did not change materially.

## CONCLUSION

In conclusion, in a cohort established in 1980 and followed up until 1999, mortality from stroke was higher in rural areas than in urban areas, especially in the case of women. This gradient remained even after adjustment for traditional risk factors. Therefore, we next need to investigate the difference in stroke incidence and case fatality between urban and rural areas.

## Appendix

List of the members of the **NIPPON DATA80** Research Group

**NIPPON DATA80:** National Integrated Project for Prospective Observation of Non-communicable Diseases And its Trends in the Aged

**Chairman:** Hirotsugu Ueshima (Department of Health Science, Shiga University of Medical Science, Otsu, Shiga)

**Consultants:** Osamu Iimura (Hokkaido JR Sapporo Hospital, Sapporo, Hokkaido), Teruo Omae (Health C&C Center Hisayama, Kasuya, Fukuoka), Kazuo Ueda (Murakami Memorial Hospital, Nakatsu, Oita), Hiroshi Yanagawa (Saitama Prefectural University, Koshigaya, Saitama), Hiroshi Horibe (Aichi Medical University, Nagakute, Aichi)

**Research Members:** Akira Okayama (The First Institute of Health Service, Japan Anti-Tuberculosis Association, Tokyo), Kazunori Kodama, Fumiyoshi Kasagi (Radiation Effects Research Foundation, Hiroshima, Hiroshima), Tomonori Okamura (Department of Preventive Cardiology, National Cardiovascular Center, Suita, Osaka), Yoshikuni Kita, Katsuyuki Miura (Department of Health Science, Shiga University of Medical Science, Otsu, Shiga), Takehito Hayakawa (Department of Hygiene and Preventive Medicine, Fukushima Medical University, Fukushima, Fukushima), Shinichi Tanihara (Department of Hygiene and Preventive Medicine, Fukuoka University, Fukuoka, Fukuoka), Shigeyuki Saitoh (Second Department of Internal Medicine, School of Medicine, Sapporo Medical University, Sapporo, Hokkaido), Kiyomi Sakata (Department of Hygiene and Preventive Medicine, Iwate Medical University School of Medicine, Morioka, Iwate), Yosikazu Nakamura (Department of Health Science Division of Epidemiology and Community Health, Jichi Medical School, Minami Kawachi, Tochigi), Fumihiko Kakuno (East-Omi Regional Promotion Bureau, Higashiomi, Shiga), Yasuyuki Nakamura (Department of Living and Welfare, Faculty of Home Economics, Kyoto Women’s University, Kyoto, Kyoto), Yutaka Kiyohara (Department of Environmental Medicine, Graduate School of Medical Sciences, Kyushu University, Fukuoka, Fukuoka), Yasuhiro Matsumura, Katsushi Yoshita (National Institute of Health and Nutrition, Tokyo, Tokyo), Hideaki Nakagawa (Department of Epidemiology and Public Health, Kanazawa Medical University, Kanazawa, Ishikawa), Hideaki Toyoshima, Koji Tamakoshi (Department of Public Health/Health Information Dynamics, Field of Social Life Science, Program in Health and Community Medicine, Nagoya University Graduate School of Medicine, Nagoya, Aichi)

**Research Associate Members:** Toshihiro Takeuchi, Mitsuru Hasebe, Fumitsugu Kusano, Takahisa Kawamoto and members of 300 public health centers in Japan, Masumi Minowa (Faculty of Humanities, Seitoku University, Matsudo, Chiba), Katsuhiko Kawaminami (Department of Public Health Policy, National Institute of Public Health, Wako, Saitama), Sohel R. Choudhury, (WHO Project Office for Non-Communicable Disease and Mental Health, Dhaka, Bangladesh), Minoru Iida (Kansai University of Welfare Sciences, Kashihara, Kashiwara Osaka), Tsutomu Hashimoto (Kinugasa General Hospital, Yokosuka, Kanagawa), Atsushi Terao (Health Promotion Division, Department of Public Health and Welfare, Shiga Prefecture, Otsu, Shiga), Koryo Sawai (The Japanese Association for Cerebro-cardiovascular Disease Control, Tokyo), Shigeo Shibata (Clinical Nutrition, Kagawa Nutrition University, Sakado, Saitama)*

*Deceased
